# Safety and evidence of CO_2_ as a vascular contrast agent as an alternative to iodine-based contrast media in vascular procedures: a systematic review by the ESUR Contrast Medium Safety Committee

**DOI:** 10.1007/s00330-025-12001-5

**Published:** 2025-09-18

**Authors:** Robert P. Wawer Matos Reimer, Peter Reimer, Andreas H. Mahnken, Marie-France Bellin, Michele Bertolotto, Torkel Brismar, Jean-Michel Correas, Katerina Deike-Hofmann, Ilona A. Dekkers, Remy W. F. Geenen, Gertraud Heinz-Peer, Carlo A. Mallio, Aart J. van der Molen, Carlo C. Quattrocchi, Alexander Radbruch, Giles Roditi, Laura Romanini, Carmen Sebastià, Fulvio Stacul, Olivier Clement

**Affiliations:** 1 Department of Diagnostic and Interventional Radiology, Faculty of Medicine and University Hospital Cologne, University of Cologne, Cologne, Germany; 2 Department of Radiology, Institute for Diagnostic and Interventional Radiology, Städtisches Klinikum Karlsruhe, Karlsruhe, Germany; 3 Department of Diagnostic and Interventional Radiology, Philipps-Universität Marburg, Marburg, Germany; 4 Department of Radiology, University Hospital Bicêtre, University Paris Saclay, BioMaps, Le Kremlin-Bicêtre, France; 5 Department of Radiology, University Hospital Trieste, Trieste, Italy; 6 Unit of Radiology, Department of Clinical Science, Intervention and Technology, Karolinska Institutet and Department of Radiology, Karolinska University Hospital in Huddinge, Stockholm, Sweden; 7 Université de Paris, AP-HP, Groupe Hospitalier Necker, DMU Imagina, Service de Radiologie, Paris, France; 8 Clinic for Diagnostic and Interventional Neuroradiology, University Clinic Bonn, German Center for Neurodegenerative Diseases, DZNE, Bonn, Germany; 9 Department of Radiology, Leiden University Medical Center, Leiden, The Netherlands; 10 Department of Radiology, Northwest Clinics, Alkmaar, The Netherlands; 11 Department of Radiology, Landesklinikum St Pölten, St Pölten, Austria; 12 Fondazione Policlinico and Research Unit Radiology, Universitario Campus Bio-Medico, Roma, Italy; 13 Centre for Medical Sciences CISMed, University of Trento, Trento, Italy; 14 Department of Radiology, Glasgow Royal Infirmary, Glasgow, UK; 15 Department of Radiology, ASST Cremona, Cremona, Italy; 16 Department of Radiology, Hospital Clinic de Barcelona, Barcelona, Spain; 17 Department of Radiology, Ospedale Maggiore, Trieste, Italy; 18 Université de Paris, AP-HP, Hôpital Européen Georges Pompidou, DMU Imagina, Service de Radiologie, Paris, France

**Keywords:** Carbon dioxide, Angiography, Contrast media, Acute kidney injury, Safety

## Abstract

**Objectives:**

This systematic review aims to analyse the different safety aspects and evidence of CO_2_ as a contrast agent in vascular applications as an alternative to iodine-based contrast media (ICM). The review addresses clinical applications, contraindications, safety measures, and the impact of CO_2_ on the risk reduction of contrast-associated acute kidney injury (CA-AKI).

**Materials and methods:**

A systematic literature search was conducted across PubMed, Web of Science, Embase, and Cochrane Library, focusing on relevant literature centred around clinical questions by the Contrast Media Safety Committee of the European Society of Urogenital Radiology.

**Results:**

Eleven studies encompassing meta-analyses, randomised controlled trials, and comparative studies were included. The review found that CO_2_ angiography is a safe alternative to ICM in various vascular applications, especially in patients at risk for CA-AKI. CO_2_ is associated with a higher incidence of minor, non-serious adverse events compared to ICM. No critical dose for CO_2_ is established, but safe administration protocols and measures were outlined. CO_2_ demonstrated a lower incidence of CA-AKI in peripheral arterial disease (PAD) procedures, but evidence in endovascular aneurysm repair (EVAR) was less conclusive.

**Conclusion:**

CO_2_ is a safe alternative to ICM in vascular procedures, potentially reducing the risk of CA-AKI, especially in PAD procedures. However, more large-scale RCTs are needed to confirm these findings and further investigate other risk factors contributing to CA-AKI in both EVAR and PAD procedures.

**Key Points:**

***Question*** *What safety aspects and evidence support CO2 use as a contrast agent in vascular applications instead of ICM*?

***Findings*** *CO2 angiography is safe when considering specific safety measures and clinical applications; evidence on the reduction of ICM volume and CA-AKI is limited*.

***Clinical relevance*** *CO2 angiography offers an alternative to ICM, especially in CA-AKI risk patients. More large-scale, multicentre RCTs are required to strengthen the evidence and to investigate other risk factors due to a high residual risk of CA-AKI when using CO2 angiography*.

## Introduction

The potential of carbon dioxide (CO_2_) as a negative contrast agent was recognised several decades ago [1]. Subsequently, based on availability, low cost, no obvious toxicity or allergies, and rapid tissue clearance, the agent was considered a natural choice as a negative contrast agent in a variety of nonvascular imaging applications such as cisternography, peritoneography, or double-contrast gastrointestinal imaging. The safety of CO_2_ over other gases is attributed to its much higher tissue solubility, minimising the risk of serious complications from inadvertent gas embolism [2].

The application for angiography was first described and further developed by Irvin F. Hawkins following an inadvertent injection of air into the coeliac trunk, followed by the exploration in several vascular territories [3–5]. CO_2_ angiography was often proposed to reduce or avoid potential adverse events of iodine-based contrast media (ICM) in patients at risk, such as those with decreased kidney function or previous allergic reactions [6, 7].

Research over the last 20 years has changed the risk assessment and safety measures in patients with severe previous allergic contrast reactions and reduced kidney function (eGFR < 45 mL/min with direct contrast injection into the renal arteries or < 30 mL/min without direct contrast injection into the renal arteries) at risk for contrast-associated acute kidney injury (CA-AKI) or contrast allergy [8–11]. Vascular applications with CO_2_ have more recently focused on diabetic patients with lower limb peripheral artery disease (PAD) with second-pass renal injection and aortic endovascular procedures with first-pass renal injection [12, 13].

The purpose of this review is to analyse the different safety aspects and evidence of CO_2_ as a contrast agent in vascular applications as an alternative to ICM. By following these guidelines, medical personnel may understand clinical applications and safely administer intravascular CO_2_, minimising the risk of complications and ensuring the effectiveness of diagnostic and interventional procedures.

## Background and imaging of CO_2_ as a vascular contrast agent

CO₂ is generally well tolerated and non-allergenic when injected into the arterial or venous systems. CO_2_ typically dissolves within the blood in 30 to 60 s [14]. When injected into an artery, CO_2_ will not pass through the capillary bed into the vein before dissolution. When injected into a vein, CO_2_ is carried directly by the blood and dissolved within the blood to the lungs, where it is eliminated within a single pass [15].

CO_2_ angiography requires time resolution with an increased DSA frame rate since the gas flows more rapidly through blood than ICM. The rapid injection may trigger spasms, causing pain at the injection site, predisposing to motion artefacts by involuntary patient movements. To address this, image mask correction and stacking software are required to maintain diagnostic image quality. For optimal imaging, the exposure rate should range from 3 to 6 frames/s. For further information, we refer to the available pertinent literature [14, 15].

## Materials and methods

For this systematic review, the literature was analysed using PubMed, Web of Science, Embase and the Cochrane Library databases from January 1956 until August 2024. Multiple searches with the following MESH terms were performed with languages limited to English and German: CO_2_ combined with angiography; contrast media; dosing biomarkers, drug; allergy and immunology; equipment safety and maximum tolerated dose, respectively. A core guideline writing group prepared eight clinical questions and converted these into PICO format [16]. The titles and abstracts were analysed for relevance and selected based on the PICO questions. The working group searched for comparative studies with strong evidence, such as meta-analyses, systematic reviews and prospective randomised controlled trials (RCTs).

A total of 2139 references were identified, of which 109 references were selected based on the abstract and title. After review of the publications and apart from narrative reviews, as well as observational studies, a total of 11 publications were selected for inclusion in this review: two meta-analyses combined with systematic reviews, one systematic review, two RCTs, two retrospective propensity score matched studies, and four retrospective comparative studies.

The concept guideline was discussed by the Contrast Media Safety Committee of the European Society of Urogenital Radiology members and consultants, revised and approved at the Contrast Media Safety Committee meeting in September 2024 in Lisbon (Portugal).

### Clinical question 1

Which vascular territories can be examined and are established clinical indications/applications?

**Fig. 1:**
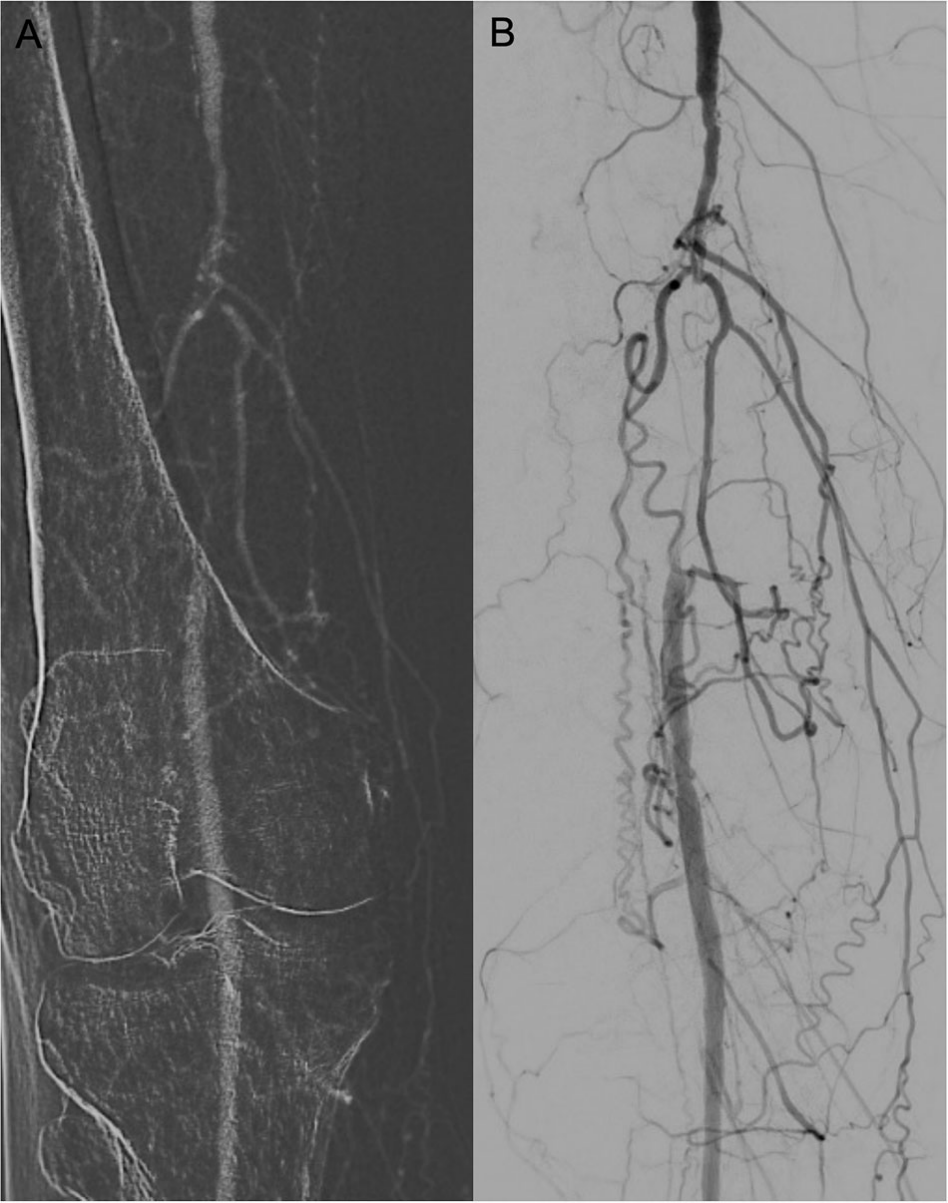
Angiography with CO₂ (**A**) and ICM (**B**) shows an occlusion of the right distal superficial femoral artery prior to revascularisation in a patient with chronic limb-threatening ischaemia (CLTI) and stage 4 chronic kidney disease (CKD)

Clinical applications are restricted to certain vascular territories (Table 1) in which CO_2_ can be used in a variety of endovascular procedures such as angioplasty, stenting, tumour embolization, embolization of vascular malformations, embolization of bleeding vessels, foreign body retrieval, catheter-directed thrombolysis/thrombectomy, trans jugular intrahepatic portosystemic shunt, trans jugular liver biopsy, or endovascular aortic repair (EVAR) (Fig. 1) [15, 17].

**Table 1:** Clinical applications and contraindications of CO_2_ angiography

**Arterial applications**
Aortogram beneath the diaphragm and intervention
Visceral arteries and intervention
Transplant renal and native renal arteries and intervention
Pelvic and femoral arteries and intervention
Bleeding and endoleak detection below the diaphragm and intervention
**Venous applications**
Upper and lower extremity venography
Haemodialysis access/shunts intervention
Vena cavography
Wedged portal venography
**Absolute contraindications**
Arteries above the diaphragm
Aortogram beneath the diaphragm in the prone position or an elevated patient’s head
Respiratory failure
Pulmonary arteriovenous malformation
Use during nitrous oxide anaesthesia
**Relative contraindications**
Pulmonary hypertension
Chronic obstructive pulmonary disease
Patent foramen ovale or septal defect

Adapted from [2, 14, 15, 17, 21]

There are various contraindications to consider (Table 1). Absolute contraindications are based on side effects in the cerebral and cardiac circulation, including applications in the thoracic aorta and brachial artery, to avoid reflux into these vessels. CO_2_ is potentially neurotoxic, causing multifocal ischaemic infarctions [18–20]. Furthermore, CO_2_ should not be injected into the abdominal aorta in the prone position or with the patient’s head in an elevated position since the buoyant gas may fill the spinal and lumbar arteries, as well as the thoracic aorta, respectively [15]. In patients undergoing nitrous oxide anaesthesia, the concurrent use of CO_2_ is contraindicated since the nitrous oxide can diffuse into the CO_2_ bubble, increasing the CO_2_ volume significantly. In the venous system, this rapid expansion of the CO_2_ bubble may result in pulmonary artery vapour lock. Relative contraindications include pulmonary hypertension and chronic obstructive pulmonary disease. CO_2_ should be used cautiously in patients with a known patent foramen ovale or atrial septal defect. However, based on current literature, screening for these conditions prior to CO₂ use is not recommended [2, 15, 17].

### Clinical question 2

What are the application techniques for safe intravascular administration of CO_2_?

During the application of CO_2_, vital signs of the patients should be monitored to detect possible adverse events using pulse oximetry, electrocardiography, blood pressure and capnography, which is the most reliable monitor for air contamination. Pulse oximetry is not an early indicator of air embolism, as oxygen saturation may remain above 90%. A drop in blood pressure within 20 s after CO₂ injection suggests air contamination in the delivery system [15, 17, 21].

There are different methods of administering CO_2_ safely, either manually or automatically. The chosen equipment setup must enable passive unidirectional flow of CO₂ from a high-pressure cylinder into a series of airtight syringes, tubing, and/or reservoir bags using a series of valves. It should allow the CO₂ to expand until it equalises with the room's atmospheric pressure while simultaneously purging room air from the system. Unlike liquid contrast agents, CO_2_ cannot be distinguished from air. Hence, contamination with air due to breached technique or equipment failure can go unrecognised. Furthermore, the catheter must be repeatedly flushed with CO_2_ prior to every CO_2_ angiogram to prevent vessel dissection from explosive delivery at high pressure. If the injection is performed manually, using a larger syringe (20–30 mL) is less likely to cause CO_2_ compression in the syringe and subsequent rapid release into the artery or organ [15, 17, 21].

**Fig. 2:**
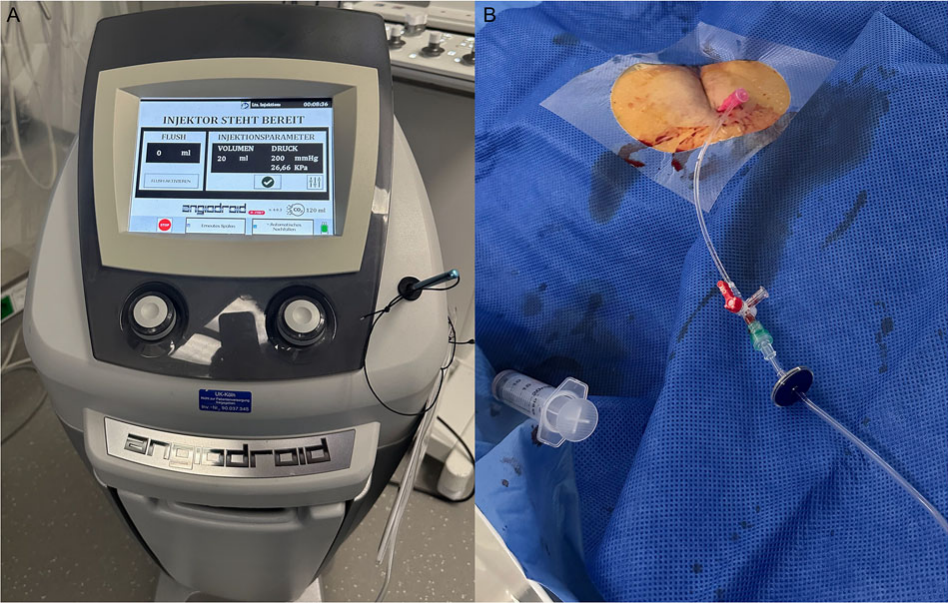
Representative image example of a CO₂ infusion setup for peripheral artery disease procedures using the Angiodroid automated digital CO₂ injector (**A**). The single-use CO₂ line can be directly connected to the sheath sidearm (**B**)

Adverse events are reported to be lower when using closed-system delivery methods. There are several automated systems available, which have not been compared with each other and with manual injection of CO_2_ regarding their safety and efficacy (Fig. 2) [2, 22]. Yet, automated injection systems should be preferred over hand-held syringe injections due to their built-in safety features, such as CO_2_ sensors, pressure regulators, volume control, and air purging mechanisms, resulting in a decreased risk of complications such as air contamination and explosive dosage [15, 17, 21, 23].

### Clinical question 3

Are there specific risk factors or side effects/complications?

Several well-described risk factors and side effects of intravascular CO_2_ have been described, with a spectrum of non-serious and potentially serious adverse events. Most adverse effects are minor and transient in duration [2].

Injection site complications are uncommon since CO_2_ is less viscous than contrast material. CO_2_ extravasation rarely occurs with an intra-arterial injection but has been observed with peripheral venous injections. Pain and discomfort may occur when CO_2_ is injected. This adverse reaction lasts less than 1–2 min, but the pain-induced motion may degrade image quality. Injection of CO_2_ into the abdominal aorta or the visceral arteries may cause epigastric pain, nausea, vomiting or diarrhoea that usually lasts a few minutes. Turning the patient from side to side usually helps relieve the symptoms [2, 15].

Serious adverse events are less common (< 1%) and comprise air contamination of the delivery system, vapour lock potentially resulting in hypoxia, ischaemia, cardiogenic shock, spinal cord injury, or neurotoxicity [21, 24]. Detailed management strategies for adverse events are discussed elsewhere [2].

### Clinical question 4

Is there a critical dose or dose equation for intravascular CO_2_?

There is no established critical dose or dose equation for the use of intravascular CO_2_. So far, only animal studies exist about critical doses of intravascular CO_2_. According to Hawkins et al, the volume of CO_2_ gas causing death of cats and dogs was 6.6 mL/kg and ≥ 500 mL, respectively. A single CO_2_ dose up to 1.6 mL/kg resulted in no changes in cardiopulmonary parameters, which corresponds to 112 mL for a 70-kg person, which is more than necessary for any clinical scenario [14]. Due to the limited data, only general recommendations exist, which propose a maximum single dose CO_2_ volume of 100 mL for angiography [17, 21]. According to a narrative review, the overall administration of unlimited volumes of CO_2_ is feasible when considering the necessary time intervals between injections [15].

### Clinical question 5

What are safe time intervals between intravascular CO_2_ applications?

Injections should be performed with specific time intervals to avoid potential serious vapour lock [17]. As a precaution for trapping (e.g., in an aneurysm), fluoroscopy of susceptible sites can be performed between CO_2_ injections. If persistent gas is visualised, positional changes can be instituted [14]. Continued visualisation of CO_2_ beyond a 3-min interval should be suspected to indicate a trapped CO_2_ bubble and/or room air contamination [17, 21]. The recommended safe intervals between intravascular CO_2_ injections generally range from 2 min to 5 min, depending on the vascular territory and patient condition. Patients with reduced pulmonary capacity, such as chronic obstructive pulmonary disease or pulmonary hypertension, tolerate smaller volumes and need longer time intervals. As there is no standard dose or interval, we recommend individualising protocols based on patient-specific factors and condition (Table 2) [17, 21]. If patients show clinical symptoms of hypoxia such as chest pain, dyspnoea, or neurological deficits, further injections should be postponed and patients oxygenated [2, 15, 17].

**Table 2:** Application settings of CO_2_ volume

Vascular territory	Volume of CO_2_ (mL)
Aorta/vena cava inferior/run-off	30–60
Visceral arteries	20–30
Pelvic arteries/veins	20–30
Femoral arteries/veins	5–20
Small arteries via microcatheter	5–10
Wedged portal venography	10–20

Adapted from [21]

### Clinical question 6

Can the amount of intravenous or intra-arterial contrast medium be reduced by adding/using CO_2_ in vascular procedures? Are there issues of the combination with ICM?

Several studies demonstrated that the use of CO_2_ angiography alone or hybrid angiography with a combination of ICM and CO_2_ may reduce the required volume of ICM. No specific issues have reported in hybrid angiography related to the combination of CO_2_ with ICM [17, 25–28].

In a two-centre RCT with a limited patient size, only 3 of 32 (9%) patients in the CO_2_ arm required an additional median volume of 10 mL ICM (range, 7–12 mL) for endovascular revascularisation of aortoiliac occlusive disease, whereas 78 mL ICM (range, 29–121 mL) was used in the ICM arm [26]. This is in line with a prospective, multicentre, observational study using a zero ICM protocol in EVAR procedures, in which only 53 patients (18.1%) required an additional injection of ICM [29]. In a retrospective, propensity-score-matched analysis with well-matched groups of 4472 patients in each group undergoing peripheral vascular interventions, the volume of ICM used was reduced by 50% (32 ± 33 vs 65 ± 48 mL; *p* < 0.01) [28]. This is in line with results of a retrospective study by Stegemann et al including 191 patients with lower limb PAD [27].

Yet, the image quality of CO2 angiography alone is lower in resolution as compared to conventional angiography with ICM and is therefore limited, particularly in smaller arteries, e.g., below the knee. Furthermore, the degree of stenosis and assessment of vessel size may be overestimated [15, 17, 30].

### Clinical question 7

Does CO_2_ affect renal function in CKD vs non-CKD patients and reduce AKI rates in EVAR procedures?

Postoperative CA-AKI may occur in up to 7.5% of cases in EVAR, explaining the importance to address this issue [31]. The application of CO_2_ in EVAR is described in review articles to decrease the volume of ICM reducing the risk of CA-AKI and may be injected through the access sheaths or endograft sheaths [2, 15, 17]. In addition, CO_2_ is proposed for the detection of endoleaks, however, with inconsistent results compared to ICM [32, 33].

The highest level of evidence data is provided by a single-centre RCT enroling 36 patients with abdominal aortic aneurysms. Patients did not show a difference in postoperative renal function. Most patients in the CO_2_ group required some ICM for contrasting the posterior iliac vessels [34].

The next highest level of evidence was a propensity score matched retrospective analysis. Patients (*n* = 34) in which EVAR was performed with CO_2_ were matched with 34 patients with similar kidney function performed with ICM selected out of 290 patients. There was no significant difference in postoperative eGFR [35].

There is a limited number of retrospective studies looking at early renal function (48–72 h) and late renal function one year following surgery in retrospective single-centre designs. Out of 322 EVAR patients a control group with an ICM load > 200 mL was selected and compared with a combined CO_2_ group consisting of CO_2_ alone (*n* = 5) and hybrid interventions with CO_2_ combined with ICM (*n* = 17) with an ICM load < 100 mL. Baseline, early and late eGFR values were not significantly different among the groups. However, eGFR values declined significantly over one year by −19.2 ± 11.1% in the group performed with > 200 mL ICM and −7.4 ± 3.5% in the hybrid group indicating that renal function post EVAR requires more detailed research and appears to be multifactorial [36].

A second retrospective analysis pooled all 321 EVAR cases over 4 years performed with either ICM (*n* = 321) or CO_2_ (*n* = 72) in two groups predisposing a bias in statistics as only 16 patients received CO_2_ alone. Radiation exposure was significantly higher in the CO_2_ group as compared to the ICM group, while postoperative eGFR decreased significantly less using CO_2_ (2.3 ± 1.1 mL/min) vs ICM (10.6 ± 5.3 mL/min) [37]. Similar results were reported in a third study with 49 patients in the ICM and 52 patients in the CO_2_ group showing a significantly higher radiation exposure, but no difference in renal function [38].

The current literature consists mainly of retrospective cohorts focusing on standard EVAR, was recently summarised in a narrative review [32]. Unfortunately, prospective comparative studies addressing standard and complex EVAR are lacking and published reports focus mainly on technical details, visibility of vessels or endoleak detection [33].

### Clinical question 8

Does CO_2_ affect renal function in CKD vs non-CKD patients and reduce AKI rates in lower limb PAD procedures?

Two meta-analyses, including systematic reviews yield the highest available level of evidence, followed by a propensity score matched analysis regarding the risk of CA-AKI between patients receiving CO_2_ and ICM undergoing peripheral vascular interventions [39, 40].

The definition of CA-AKI varied across the studies, with most using a > 25% rise or > 0.5 mg/dL in serum creatinine within 48–72 h post-procedure [28, 39, 40]. One meta-analysis reviewed eight studies (one RCT and seven observational studies) involving 677 patients who underwent 754 procedures. The studies ranged from 1995 to 2015, with 185 patients in the CO_2_ group and 492 in the ICM group. Six studies reported a lower incidence of AKI in the CO_2_ group (overall, 4.3%) compared to the ICM group (overall, 11.1%). The corresponding pooled odds ratio favoured CO_2_ (OR 0.465; *p* = 0.048). In a subgroup analysis of patients with chronic kidney disease (CKD), there was no statistically significant difference in AKI incidence between CO_2_ and ICM (4.1% vs 10.0%; OR 0.449; *p* = 0.117). However, the sample size for this subgroup was small (*n* = 349). Furthermore, CO_2_ was associated with a higher incidence of minor, non-renal adverse events, including limb and abdominal pain, nausea, and vomiting, compared to ICM. There was no difference in mortality [39].

In the other meta-analysis, eight studies (three RCTs and five cohort studies) involving a total of 1128 patients were included with two studies focusing on patients with CKD stage 3 or higher. This meta-analysis showed that CA-AKI event rates were lower in participants receiving CO_2_ compared to those receiving ICM (8.6% vs 15.2%; relative risk (RR) 0.59). The risk reduction was more pronounced in the RCTs (4.1% vs 13.4%; RR 0.33) compared to cohort studies (10.8% vs 15.6%; RR 0.78). When including only studies with low or moderate risk of bias, CO_2_ still showed a statistically significant lower risk of CA-AKI compared to ICM (8.8% vs 18%; RR 0.55). For patients with GFR < 60 mL/min (CKD stage 3), the risk reduction was RR 0.69, but there remained a high residual risk, suggesting the influence of other risk factors [40].

The results align with the more recent retrospective, PS-matched analysis, which included 4472 patients in each group. Peripheral vascular interventions using CO_2_ angiography showed lower rates of CA-AKI (3.9% vs 4.8%; *p* = 0.03). Additionally, the use of low ICM volumes in CKD patients was associated with a reduced the risk of CA-AKI (hazard ratio, 0.59; *p* < 0.01) [28].

## Discussion

The evidence in the literature on the safety and benefits of CO_2_ angiography as an alternative to ICM is limited due to the small number of meta-analyses, systematic reviews, RCTs and retrospective comparative studies. CO_2_ angiography seems to be a safe alternative to ICM when considering the described clinical applications, contraindications, safety measures and the higher incidence of non-serious adverse events.

The reviewed literature suggests that CO_2_ angiography has the potential to reduce the volume of ICM in EVAR and lower limb PAD procedures [26–28]. Concerning the benefits of CO_2_ angiography over ICM regarding the reduction of CA-AKI in EVAR procedures, the level of evidence was low as only one RCT and several retrospective studies exist. The available data suggest that CO_2_ may possibly reduce the risk of CA-AKI as compared to ICM, which remains inconclusive due to small sample sizes and variability in study designs [34–38]. More evidence exists about the benefits of CO_2_ angiography over ICM regarding the reduction of CA-AKI in lower limb PAD procedures, as two meta-analyses including systematic reviews of RCTs and cohort/observational studies, as well as one propensity score matched study, provided a moderate level of evidence. The findings suggest that CO_2_ angiography may reduce the incidence of CA-AKI in lower limb PAD procedures, irrespective of CKD stage, compared to ICM angiography, while further research is needed for patients with advanced CKD [28, 39, 40].

However, there was a high residual risk of CA-AKI in EVAR and lower limb PAD procedures using CO_2_ angiography, indicating other contributing factors to CA-AKI, such as hypertension, heart failure, coronary artery disease, diabetes or microembolic renal events during the cannulation of the renal arteries in branched, chimney or fenestrated EVAR procedures [39–42]. The risk of a functional decline appears to be more complex than the simple question of the contrast used. There are several reports addressing thromboembolic emboli, cholesterol emboli or catheter material embolizing from the surface, especially when a hydrophilic coating is utilised [43–45]. Hence, more research concerning other potential risk factors, as well as further large-scale RCTs investigating the potential benefits of CO_2_ angiography over ICM on the risk reduction of CA-AKI in EVAR and PAD procedures, is needed.

To conclude, the use of CO_2_ in vascular procedures seems to be a safe alternative to ICM when paying attention to specific safety measures and appears to reduce the volume of ICM needed. The level of evidence for reducing the risk of CA-AKI was low for EVAR procedures and moderate for PAD procedures. More large-scale, multicentre RCTs are required to strengthen the evidence and to investigate other risk factors due to a high residual risk of CA-AKI.

## Abbreviations

CA-AKI: Contrast-associated acute kidney injury
CO_2_: Carbon dioxide
EVAR: Endovascular aortic repair
ICM: Iodine-based contrast media
PAD: Peripheral artery disease
RCT: Randomised controlled trial
